# Classroom Misbehavior in the Eyes of Students: A Qualitative Study

**DOI:** 10.1100/2012/398482

**Published:** 2012-07-31

**Authors:** Rachel C. F. Sun, Daniel T. L. Shek

**Affiliations:** ^1^Faculty of Education, The University of Hong Kong, Hong Kong; ^2^Department of Applied Social Sciences, The Hong Kong Polytechnic University, Hong Kong; ^3^Public Policy Research Institute, The Hong Kong Polytechnic University, Hong Kong; ^4^Kiang Wu Nursing College of Macau, Macau; ^5^Division of Adolescent Medicine, Department of Pediatrics, University of Kentucky College of Medicine, Lexington, KY 40506, USA; ^6^Department of Social Work, East China Normal University, Shanghai 200062, China

## Abstract

Using individual interviews, this study investigated perceptions of classroom misbehaviors among secondary school students in Hong Kong (*N* = 18). Nineteen categories of classroom misbehaviors were identified, with talking out of turn, disrespecting teacher, and doing something in private being most frequently mentioned. Findings revealed that students tended to perceive misbehaviors as those actions inappropriate in the classroom settings and even disrupting teachers' teaching and other students' learning. Among various misbehaviors, talking out of turn and disrespecting teacher were seen as the most disruptive and unacceptable. These misbehaviors were unacceptable because they disturbed teaching and learning, and violated the values of respect, conformity, and obedience in the teacher-student relationship within the classroom. The frequency and intensity of misbehaviors would escalate if students found it fun, no punishment for such misbehaviors, or teachers were not authoritative enough in controlling the situations. Implications for further research and classroom management are discussed.

## 1. Introduction

There are numerous studies examining the definitions and range of student misbehaviors. For example, in the United Kingdom and Australia, researchers defined classroom misbehaviors as behaviors which are disruptive to classroom order and cause trouble to teachers, such as making nonverbal noise, disobedience, talking out of turn, idleness/slowness, nonpunctuality, hindering others, physical aggression, untidiness, out of seat, and verbal abuse [1–3]. In the United States, James [4] conceived students misbehaved when they “either did what they were not supposed to do or did not do what they were supposed to do” (page 9), ranging from fooling around as mild misbehavior to fighting as severe misbehavior. In the Caribbean contexts, student misbehaviors in classroom included those disruptive behavior which hampered teaching, and learning, such as classroom disconformity, verbal and physical hostility, defiance of authority, task avoidance, inappropriate use of school property, inconsiderate interpersonal relationships, over-reactions to normal situations, and technological related factors [5].

While classroom misbehavior is generally interpreted as disruptive and improper behavior that adversely affects the order, teaching, and learning in classroom, it is noteworthy that the range of student misbehavior varies across cultures [6, 7]. Particularly, as respect for authority, conformity, and obedience are highly valued in the Chinese school context [8], some student behaviors would be considered as problematic or unacceptable in Chinese classroom but not elsewhere. For example, in the traditional Chinese culture, students who kept on asking questions would be regarded as “troublesome” students whereas students strictly followed teachers' orders were regarded as excellent students. However, in contrast to the studies conducted in the Western cultural contexts, there have been very limited research findings on student misbehavior in the Chinese cultural contexts [9, 10], particularly in Hong Kong [11, 12]. Therefore, it is necessary to understand more about the definition and conception of student misbehavior in Hong Kong. This need is particularly acute when we realize that adolescent behavior has changed tremendously with the advance in technology. Through the Internet, it does not take long to popularize certain misbehavior in young people.

Against the above background, a recent study was conducted in Hong Kong Chinese schools by Sun and Shek [13], which showed that most of the classroom misbehaviors reported by the teachers included doing something in private, talking out of turn, verbal aggression, disrespecting teachers, nonattentiveness/daydreaming/idleness, sleeping, habitual failure in submitting assignments, and out of seat. These findings suggest that classroom misbehaviors can be defined as those behaviors that involve rule breaking, violating the implicit norms or expectations, being inappropriate in the classroom settings and upsetting teaching and learning. The findings also matched with the categorization of misbehavior as off-task, disruptive, and unruly behaviors [14]. Off-task behaviors like doing things irrelevant to the class learning, or daydreaming and sleeping, are regarded as classroom misbehaviors. These misbehaviors would become disruptive if their frequency and intensity escalated. Similar to those obvious disruptive behaviors such as talking out of turn and out of seat, they impede teachers' teaching and students' learning. Failing one's responsibility in handing homework on time and lacking respect to classmates and teachers by showing verbal and physical aggressiveness are definitely breaking the conventional rules and values in Chinese classroom. Among the various forms of misbehaviors, “talking out of turn” was constantly rated by teachers as the most frequent and troublesome misbehavior across contexts [15]. However, it is doubtful whether behaviors considered as problematic, inappropriate, disturbing, or unruly in the eyes of teachers are necessarily shared by the students.

One serious limitation of the research on student misbehavior is that most of the existing studies on school misbehavior were primarily based on teachers' perceptions and ratings, (for example, [1, 9, 11, 12]). However, it can be criticized that teachers usually have a dissimilar conception of school misbehavior with their students due to differences in social roles and values [16]. Moreover, teachers and students might have different degree of tolerance in judging whether a particular action is a misbehavior or not, or in rating the intensity of disruptiveness on the same misbehavior [17]. Hence, it is argued that findings simply based on teachers' responses might be partial or biased, and the perceptions of students should also be included. Nevertheless, there is scant research studies investigating students' perceptions of classroom misbehavior [4, 18]. Although a study was conducted in Hong Kong to examine misbehavior from the students' perspective [19], it focused on students' explanations of their school misbehavior and effective means to deal with student misbehavior. However, it can be argued that any meaningful intervention would not be possible if students' conceptions and definitions of classroom misbehavior are not thoroughly examined before the intervention. Thus, the present study attempted to examine classroom misbehavior from the students' point of views, and to understand what are the most common, disruptive, and unacceptable misbehaviors in the eyes of students.

The overarching goal of this study was to examine classroom misbehavior from the perspective of students in junior secondary school settings in Hong Kong. In this study, classroom misbehavior was regarded as a kind of problem behavior [20–22]. It is a descriptive and exploratory qualitative research study which attempted to identify and categorize classroom misbehaviors reported by a group of Grade 7 to 9 students. By understanding the issue from the students' perspective, the present findings would contribute to the existing literature and shed light on teaching, discipline, or guidance work in the school context.

A qualitative research method was adopted in this study. This method can enrich our understanding of the problem area because most of the studies in this area are quantitative in nature. By listening to the voices of the students, it is expected that the findings can help generate findings that cannot be adequately captured by those based on the teachers. A general qualitative study orientation (i.e., no particular qualitative research strand was adhered to) was adopted, with the following elements intrinsic to the study. First, voices of the students instead of the “experts” or “adults” were heard. Second, narratives of the students were focused upon. Third, individual interviews were conducted in nonartificial setting. As it is an exploratory study, a general qualitative orientation close to a postpositivistic tradition (qualitative data collection with coding and thematic analyses) was sufficient for this purpose.

## 2. Methods

### 2.1. Participants

The informants were 18 junior secondary school students from three schools, with each school admitting students having low, medium, or high academic competencies. In each school, six students (one boy and one girl in Grade 7, Grade 8, and Grade 9) were randomly selected by their teachers and they were invited to join an individual interview on a voluntary basis. The informants comprised nine boys and nine girls, with a mean age of 13.9 years old (range = 12–17 years old). Although there is no “sacred number” in qualitative research, an engagement of 18 participants could be regarded as on the high side. Also, recruitment of students from schools with different academic abilities and gender could ensure that a wide range of experiences would be examined. Written consent from the school principals and the informants, as well as passive parental consent from the student informants, were obtained prior to data collection. At the beginning of each interview, anonymity and confidentiality of the study were clearly explained to the informants. Before conducting this research, ethical approval was obtained from the Human Research Ethics Committee, The University of Hong Kong.

### 2.2. Instruments

A self-constructed semi-structured interview guide was used for each individual interview. In the interview guide, questions and prompts were used to explore the informants' perceptions of students' problem behaviors and teachers' management strategies in the classroom and school contexts. The informants were asked to define “problem behaviors” based on their own understanding and interpretation. They were invited to use real-life examples to further illustrate their views. The average time for an interview was 48 minutes (range = 33–71 minutes). Each interview was conducted by two trained interviewers in Cantonese (the mother tongue of both the interviewers and interviewees). The interviews were audio-taped with informants' prior consent and transcribed in verbatim after the interviews.

 As many open-ended questions were covered in the interview guide, only data related to the following questions were analyzed in this paper. Interested readers can write to the first author to obtain the full list of interview questions.

1. In the classroom, what student problem behaviors are there? Please list out as many as possible and describe them.
2. Among these problem behaviors, which one(s) is/are the most common?
3. Among these problem behaviors, which one(s) is/are the most disruptive to teaching and learning?
4. Among these problem behaviors, which one(s) is/are the most unacceptable? Please illustrate.

### 2.3. Data Analysis

The data were analyzed by general qualitative analyses techniques [23], in which codes and categories of misbehavior were inductively derived from the data. A colleague who has a Bachelor degree in psychology and teaching experiences conducted the first-level coding to cluster semantically similar words, phrases, and/or sentences that formed meaningful units in each conclusion at the raw response level. The first author further checked and carried out second-level coding and categorization, in which similar codes were grouped to reflect higher-order categories of themes. The coding and categorization were finalized with consensus among the coders, and agreed by another colleague with a Bachelor degree in psychology and professional counseling training.

The researchers were aware of their possible biases in their conceptions of student misbehavior because they had worked in the education field for some time. Therefore, checking procedures were carried out to look at the consistency in the coding process without the involvement of the authors. Both intra- and interrater reliability on the coding were calculated to ensure the credibility of the findings. Intrarater reliability tests were conducted by the two coders independently, whereas interrater reliability tests were conducted by two colleagues (one has a Master degree and several years of teaching experience and one has a Bachelor degree) independently. In each reliability test, 20 raw responses were randomly selected for each rater to code without referring to the original codes. Results of the reliability analyses were on the high side: intrarater agreement percentages were both 100% for both coders; interrater agreement percentages were 80% and 90% for each coder when they coded the analyses of the counterpart. To enhance the quality of the research, audit trails were developed and data analyses processes were systematically documented.

## 3. Results

Table 1 summarizes the categorization of responses based on students' perceptions of problem behaviors inside classroom reported by 18 student informants. The 107 responses could be classified into 19 main categories and six of them could further be divided into subcategories. The frequently reported classroom misbehaviors were “talking out of turn”, “disrespecting teachers”, “doing something in private”, “verbal aggression”, “out of seat”, “sleeping”, “playing”, “clowning/making fun”, “(habitual) failure in submitting assignments”, “non-attentiveness/looking out of window”, and “non-verbal communication”. Among them, “talking out of turn” and “out of seat” were viewed as the most common misbehavior in the classroom. “Talking out of turn” and “disrespecting teachers” were rated as the most disruptive and unacceptable problem behaviors.

### 3.1. Talking Out of Turn

The informants perceived that students usually talked out of turn, such as *“do not put up their hands before answering questions” *and* “shout the answer out”* (Student A05). This kind of calling out, as well as asking nonsense questions without teacher permission, were regarded as disturbing. As mentioned by Student B08:

*“No one likes to hear people speaking too loudly. It will affect the learning environment. The class is often distracted by this kind of noise. Also the noise will largely affect each student psychologically. I mean student may be annoyed by the noise. They will become more agitated, easy to lose their temper and becomes inattentive in class. It is fine if you make noise but you should not disturb others”*.

They also revealed that “conversation among students” was the most common and annoying. Student B10 described:

*“When the teacher is teaching, students at the back talk to each other… Sometimes they are not too excessive, but sometimes they speak too loudly that we can hardly hear what the teacher is saying… There are not just two (students) but sometimes a cluster… just like to kick up a fuss, because sometimes you won't sit next to your friend. Your friend may sit far away from you at the diagonal corner. You have to speak out loudly in order to let your friend hear you. Then, other students will hear you and all of them will laugh together. This is like ripple effect”*.

“Talking out of turn”, especially chatting among students, was perceived as most disruptive to teachers' teaching and students' learning. Student C10 explained:

*“Chatting will disturb teaching. If they chat very loudly or do not listen to the teacher, they and other students will miss some new knowledge. Also the teacher may think that you do not have motivation to learn which may make him/her unhappy”*.

It was perceived as unacceptable when the misbehavior becomes so noisy and uncontrollable that it adversely affects other students' learning. Student A10 revealed*:*

*“It is acceptable if you chat in a low voice. But the point is you chat louder and louder despite being asked to stop. This is the most distracting behavior which makes others unable to concentrate in class”*.

### 3.2. Disrespecting Teacher

Behaviors that were disrespectful to teachers, such as disobedience, refusing to follow instructions, rudeness, talking back, arguing with teacher, offending, or attacking teachers, were reported as an obvious problem behavior in the classroom. Student B08 described how students used some subtle ways to offend their teachers:

*“The students do not respect their teacher. Sometimes they do not treat their teacher as a person. Generally speaking, they do not care about him/her. They may pretend to be good, but in fact, they behave differently at the back of their teacher”*.

On the other hand, some students would attack teachers directly. Student A06 recalled:

*“Such as our class teacher, when teaching, some boys offended him/her for no reason. It is because the teacher does not know how to scold the students. That's why those boys like to assault him/her”*.

Arguing with teachers could disrupt teaching and learning because it was time consuming. Student C07 commented that *“if the teacher scolds us, we will argue back, and then the teacher will scold us even much more. It uses up all the time”*. Student B08 also considered it as an unacceptable behavior: “*I think politeness of a student is very important. Sometimes if the teacher asks you to do something, you need to show your politeness in addition to respect… A person's virtue is more important than his/her knowledge”*.

### 3.3. Doing Something in Private

Students liked to do something unrelated to classroom learning, such as doing homework of other subjects, dealing with personal stuff, having irrelevant drawing, or using mobile phone. However, not all informants would regard “doing something in private” as a kind of problem behavior. For example, Student C09 explained: *“some students use mobile phone to text when the teacher is not looking at them… Actually, I think using mobile phone or pushing classmates are not problematic. It will not affect the learning atmosphere… playing mobile phone only affects the individual…a person's learning attitude… and usually the teacher do not see them so that it affects nothing”*.

### 3.4. Out of Seat and Sleeping

The informants also pointed out that “out of seat” (including changing seats and wandering around the classroom) and “sleeping” were other problem behaviors in the classroom. Moreover, these problem behaviors would become more serious and spread over if without proper teacher control. Some students also considered that both of these behaviors would affect classroom teaching and learning. As two students described:

*“The teacher sometimes is not aware of students who are out of seat, and also he/she may be dealing with the students who are making noise…so he/she is not able to handle those who leave their seats”*.  (Student A06)

*“When the students, who are very tired but try to endure the sleepiness, find their classmate is sleeping, they will begin to lay on the table, sleep or do other things because they realize that the sleeping student will not be punished, that means they are allowed to do so”*.    (Student A09)

### 3.5. Verbal Aggression and Physical Aggression

“Verbal aggression” (including attacking classmates, quarrelling with classmates, speaking foul language, teasing classmates, and gossiping) and “physical aggression” (including striking, attacking and pushing classmates, and destroying things) were reported as problem behaviors. Student might feel bad and even threatening when there was hostility. As Student C09 expressed, *“I feel hurt when I saw my classmate was struck by others… We are classmates, we are friends… I don't dare to stop them because I'm afraid that they will strike me too”*.

### 3.6. Other Forms of Misbehaviors

As shown in Table 1, there were other problem behaviors reported by the informants. They were “playing”, “clowning/making fun”, “failure in submitting assignments” (and in a habitual manner), “nonattentiveness” (also including looking out of window), “nonverbal communication” (via body language or passing papers), “making noise” (like rocking chair and singing), “isolating classmates”, “copying homework”, “forget to bring textbook and other learning materials to class”, and “disturbing other classmates” (e.g., pulling classmate's braid, tickling others, messing up other's things). Individual informants also reported that “invasion of privacy” (tried to sneak a quick look of other personal stuffs) and “intimate physical contact” (likes touching and hugging during class) were problem behaviors in the classroom.

## 4. Discussion

The present study attempted to examine classroom misbehaviors perceived by junior secondary school students in Hong Kong. A total of 19 problem behaviors were mentioned by the students, including talking out of turn, disrespecting teachers, doing something in private, verbal aggression, out of seat, sleeping, playing, clowning/making fun, (habitual) failure in submitting assignments, nonattentiveness/looking out of window, nonverbal communication, physical aggression, isolating classmates, making noise, copying homework, forget to bring textbook and other learning materials to class, disturbing other classmates, invasion of privacy, and intimate physical contact (see Table 1). The present findings showed that many of the misbehavior categories are similar to those reported in the studies conducted in the Western and Chinese cultural contexts [1, 9, 11], and they are consistent with those reported by teachers and students as well [4, 13]. The findings generated from the Chinese students' perspective lent support to the previous research findings that “talking out of turn” is the most common and disruptive misbehavior inside the classroom [15].

In conjunction with the previous study conducted by the authors [13], the present study showed that the views of both the teachers and students were complementary in understanding the definition and types of student misbehaviors inside classroom. In terms of the categorization of the classroom misbehavior, there was a consensus in some of the misbehavior, though some differences were also identified. While teachers perceived lateness to class, eating/drinking and passive engagement in class were problem behaviors, students did not regard these to be misbehaviors. On the other hand, while students reported that disturbing classmates, intimate physical contact, invasion of privacy, isolating classmates, and making noise were problem behaviors, their teachers did not mention these behaviors in their narratives.

There are two explanations for the discrepancies in the conceptions of misbehavior between teachers and students. First, some misbehaviors may be more easily identified among students than by teachers such as those misbehaviors performed at the back of the teacher inside the classroom. It was mentioned by the students that teachers were not aware of some misbehaviors when they were concentrated in teaching or dealing with other problem behaviors in the classroom. Second, the discrepancies might be due to different levels of tolerance between the students and teachers. For example, some students did not perceive some off-task behaviors as problematic as they considered that these behaviors would not cause disturbances to others. Moreover, students and teachers might view the same thing through different lens. For example, students who had not brought textbook to class were perceived as “forgetfulness” in the eyes of the students but perceived as “unprepared for learning” by the teachers. Both “forgetfulness” and “unpreparedness” refer to a lack of responsibility in the expected role of students, but the level of accusation for “unprepared for learning” seemed to be more serious than that for “forgetfulness”. Obviously, the present study shows that collecting students' views can help provide a more comprehensive picture in describing various types of student misbehaviors.

In the present findings, all the reported misbehaviors were actually off-task and inappropriate behaviors inside classrooms. This observation is in line with the assertion that misbehavior is behavior “students either did what they were not supposed to do or did not do what they were supposed to do” [4, page 9]. It is noteworthy that some of these misbehaviors are disruptive to teaching and learning as well. For instance, asking nonsense questions and fighting with teachers are wasting the time which is timetabled for valuable learning. Students who are running out of seat and playing would disturb others. Students would learn nothing if they fell asleep in class, and the worse was more students would slumber as a result of imitation. Interestingly, some misbehaviors, such as chatting in a low voice and doing irrelevant things in private, were perceived as nonproblematic as they simply affected one's own learning and did not disturbing other students', or when these behaviors were not detected by the teachers and thus did not disturb teachers' teaching. This observation may be due to the fact that contemporary young people have become more egocentric (i.e., not really caring about others' feelings) and pragmatic (i.e., less emphasis on moral principles).

Among various misbehaviors reported in this study, both talking out of turn and disrespecting teachers were rated as the most unacceptable problem behaviors. Obviously, these behaviors, particularly if uncontrollable, are disruptive to classroom learning and thus unacceptable. Moreover, it is interesting to note that some students found these misbehaviors as intolerable, when they upheld the personal virtues of politeness and respect, and the Chinese values of conformity and obedience, in the teacher-student relationship within the school context [8]. Therefore, they regarded misbehaviors as those behaviors that were impolite, challenging, noncompliant, and rebellious behaviors because they violated the hierarchical teacher-student relationship as well as the order and organization of the classroom [24]. Also, attacking and striking classmates, though rarely happened, were unacceptable because they upset the harmonious peer relationship and classroom atmosphere. All these misbehaviors would elicit negative emotions, such as annoying, hurtful, and even threatening, that in turn affected learning adversely.

Some students also mentioned that the frequency and intensity of misbehaviors, such as chatting, sleeping, and out of seat, would escalate if they found it fun, or no punishment for such misbehaviors, or teachers were not authoritarian enough in controlling the situations. Dreikurs [25] stated that student misbehavior is a purposeful endeavor to gain social recognition, while Glasser [26] stated that student misbehavior is a response to the classroom context or instruction that cannot satisfy their basic needs of love, belongingness, self-worth, freedom, fun, and survival. Thus, misbehavior usually occurs when there is a mismatch between the school and student needs [27]. It was suggested that having caring teachers who are willing to cater for students needs might be one of the helpful means to deal with student misbehavior [19]. Research findings also showed that a combination of care and behavioral control [28], schoolwide/whole-school positive behavior support [29, 30], character education [31], social skills training [32], and positive youth development programs [33–35] was effective in mitigating students' problem behavior. In particular, positive youth development programs such as the Project P.A.T.H.S. would help to reduce misbehavior in class. The existing evaluation findings showed that this program was able to promote psychosocial competencies (which may eventually lower classroom misbehavior) and reduce adolescent delinquency [36–39].

The present findings underscore the importance to view student misbehavior through the lens of students. Practically, they shed lights on managing student behavior and enhancing student learning and development via identifying students' needs and matching up with the classroom context. It is equally important for future research to further explore the reasons behind student misbehaviors and the effective means of managing student behaviors from both students' and teachers' perspectives. As mentioned above, there are few studies looking at both the perspectives of the teachers and students. Theoretically, it is important to look at the discrepancies between teachers and students on student misbehavior and understand how such differences may affect school policies on school discipline and counseling. For researchers adopting an interpretive perspective, the social reality is fluid in nature. Hence, it is important to look at things from different angles and hear voices of different parties. For critical theories, it is even more important to understand the views of different stakeholders so that we can empower them.

There are several limitations in this study. First, it was a small-scale exploratory study with 18 students from three secondary schools recruited via convenience sampling. Hence, representativeness of the findings should be viewed with caution. However, it is noteworthy that the informants were randomly selected from the students. Second, as the informants were junior secondary school students, generalization of the findings to other age groups, like upper secondary or elementary school students, needs further validation. Third, only a one-shot interview was conducted for each informant. It would be ideal if more interviews over a longer period of time can be conducted. Finally, it may be criticized that the students may share the ideologies of the teachers. However, as the students were randomly selected, this possibility is not too high. Regardless of these limitations, this study is a good endeavor to understand the issue of classroom misbehavior from the perspectives of students, which helps to give a fuller picture of the phenomenon of classroom misbehavior, particularly in Hong Kong Chinese school context.

To what extent the present study is an acceptable qualitative study? Based on the criteria proposed by Shek et al. [40] to evaluate the quality of qualitative research, the present study can be regarded as having good quality. First, there was an explicit statement of the philosophical base of the study (Criteria 1). Second, the number and nature of the participants of the study were justified (Criteria 2). Third, the data collection procedures were given in details (Criteria 3). Fourth, biases and preoccupations of the researchers were discussed (Criteria 4) and how such biases were handled (Criteria 5) are described. Sixth, interrater reliability and intrarater reliability procedures were used (Criteria 6) and the present findings were triangulated with those collected from the teachers (Criteria 7). Seventh, the researchers were consciousness of the importance and development of audit trails (Criteria 9). Eighth, alternative explanations for the observed findings were discussed (Criteria 10). Ninth, negative evidence were accounted for (Criteria 11). Finally, limitations of the study were examined (Criteria 12). Because of time and manpower constraints, the researchers were not able to include peer checking and member checking procedures (Criteria 8), which should be carried out in future studies.

## Figures and Tables

**Table 1:** A Summary of students' perceptions of student problem behaviors inside classroom (*N* = 18).

Category	Subcategory	Number of responses	Number of responses regarding on the most common problem behavior	Number of responses regarding on the most disruptive behavior	Number of responses regarding on the most unacceptable problem behavior
Talking out of turn	Asking nonsense question	1	0	1	0
	Calling out	5	1	1	0
	Having disruptive conversation	15	6	9	3

	Subtotal	21	7	11	3

Disrespecting teacher	Disobedience/Refusing to carry out instructions	4	0	2	1
	Rudeness/Talking back/Arguing with teacher	4	1	2	3
	Offending/Attacking teacher	3	0	0	0

	Subtotal	11	1	4	4

Doing something in private	Dealing with personal stuff	4	0	0	0
	Doing homework	3	0	0	0
	Using electronic device (texting, playing games, surfing webpages, listening to music)	1	2	0	0
	Irrelevant drawing	2	0	0	0

	Subtotal	10	2	0	0

Verbal aggression	Attacking classmates	2	0	0	0
	Gossiping	2	0	0	0
	Quarrelling with classmates	1	0	0	0
	Speaking foul language	0	0	0	1
	Teasing classmates	3	0	0	0

	Subtotal	8	0	0	1

Out of seat	Changing seats	1	0	0	0
	Wandering around the classroom	6	4	2	1

	Subtotal	7	4	2	1

Sleeping		7	0	2	0
Playing		6	0	1	0
Clowning/Making fun		5	2	2	0
(Habitual) failure in submitting assignments		5	1	0	0
Non-attentiveness/Looking out of window		5	1	1	0
Non-verbal communication	Via body language, papers	5	0	1	1
Physical aggression	Attacking classmates	1	0	1	1
	Destroying things	1	0	0	0
	Pushing classmates	1	0	0	0
	Striking classmates	1	1	0	1

	Subtotal	4	1	1	2

Isolating classmates		3	0	0	1
Making noise	E.g., rocking chair, paper-playing, singing	2	1	0	0
Copying homework		2	0	0	0
Forget to bring textbook and other learning materials to class		2	1	0	0
Disturbing other classmates		2	0	0	0
Invasion of privacy		1	0	0	0
Intimate physical contact		1	1	0	0

Total responses		107	22	25	13
